# Age-Adjusted Mortality Rates and Age and Risk–Associated Contributions to Change in Heart Disease and Stroke Mortality, 2011-2019 and 2019-2020

**DOI:** 10.1001/jamanetworkopen.2022.3872

**Published:** 2022-03-23

**Authors:** Stephen Sidney, Catherine Lee, Jennifer Liu, Sadiya S. Khan, Donald M. Lloyd-Jones, Jamal S. Rana

**Affiliations:** 1 Division of Research, Kaiser Permanente Northern California, Oakland; 2 Department of Preventive Medicine, Northwestern University Feinberg School of Medicine, Chicago, Illinois; 3 Division of Cardiology, Department of Medicine, Northwestern University Feinberg School of Medicine, Chicago, Illinois; 4 Department of Cardiology, Permanente Medical Group, Oakland, California; 5 Department of Medicine, University of California San Francisco

## Abstract

This cohort study examines the association of age and underlying disease risk with change in heart disease and stroke mortality from 2011 to 2020.

## Introduction

While the age-adjusted mortality rate from heart disease (HD) and stroke decreased between 2011 and 2019, the number of deaths due to HD and stroke increased in association with the rapid growth rate of the population aged 65 years and older.^1^ From 2019 to 2020, the estimated age-adjusted mortality rate increased by 15.9%, largely due to COVID-19 mortality,^2^ with increases in age-adjusted mortality from HD (4.3%) and stroke (6.4%). We examined relative contributions of aging vs underlying disease risk in the total population and in major race and ethnicity groups.

## Methods

The institutional review board of Kaiser Permanente Northern California determined this cohort study to be exempt from review and informed consent because it used a deidentified public-use data set. This study is reported following the Strengthening the Reporting of Observational Studies in Epidemiology (STROBE) reporting guideline.

Age-adjusted mortality rates for HD and stroke, age-specific numbers of deaths, and population estimates from 2011 to 2020 were determined from the Centers for Disease Control and Prevention Wide-Ranging Online Data for Epidemiologic Research (WONDER) database.^2^ Using the method of Bashir et al,^3^ year-to-year change in age-associated deaths was estimated by multiplying the age-specific death rate for 1 year by the age-specific population of the next year. The sum of the products represented age-associated change in deaths. Risk-associated mortality was calculated as change in total deaths minus age-associated change in deaths, representing deaths associated with underlying changes in disease risk. The US Census was the source of self-reported race and ethnicity data, which were included to enable examination of mortality outcomes.

## Results

**Figure 1.**
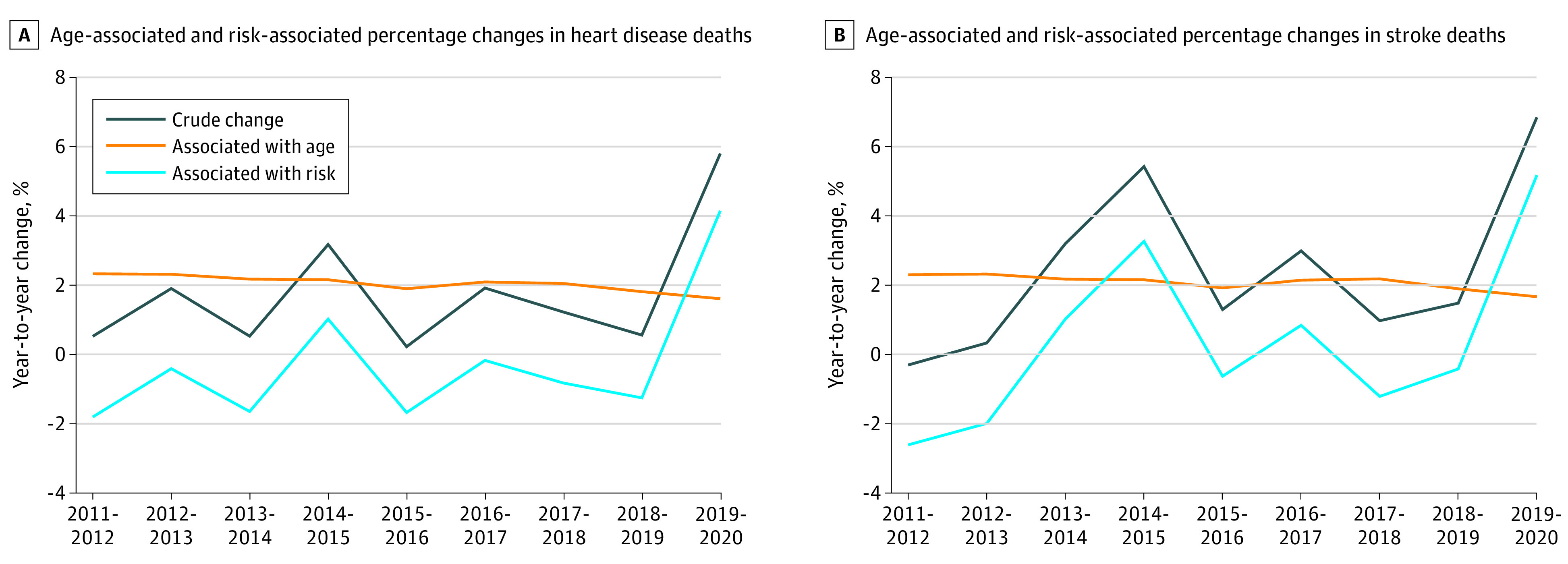
Age- and Risk-Associated Change in Heart Disease and Stroke Deaths

From 2011 to 2019, the number of HD deaths increased from 596 577 to 659 041 deaths (10.4%) and stroke deaths increased from 128 932 to 150 005 deaths (16.3%). The total age-associated increase in HD deaths from 2019 to 2020 was 104 784 deaths (17.6%), while the risk-associated decrease was 42 326 deaths (7.1%). For stroke, the total age-associated increase was 23 371 deaths (18.1%), while the total risk-associated decrease was 2301 deaths (1.8%). From 2019 to 2020, HD and stroke deaths increased by 37 934 deaths (5.8%) (Figure 1A) and 10 262 deaths (6.8%) (Figure 1B), respectively. The age-associated increases were 10 599 deaths (1.6%) and 2502 deaths (1.7%) for HD and stroke, respectively, while the risk-associated increases were 27 335 deaths (4.1%) for HD and 7760 deaths (5.2%) for stroke.

**Figure 2.**
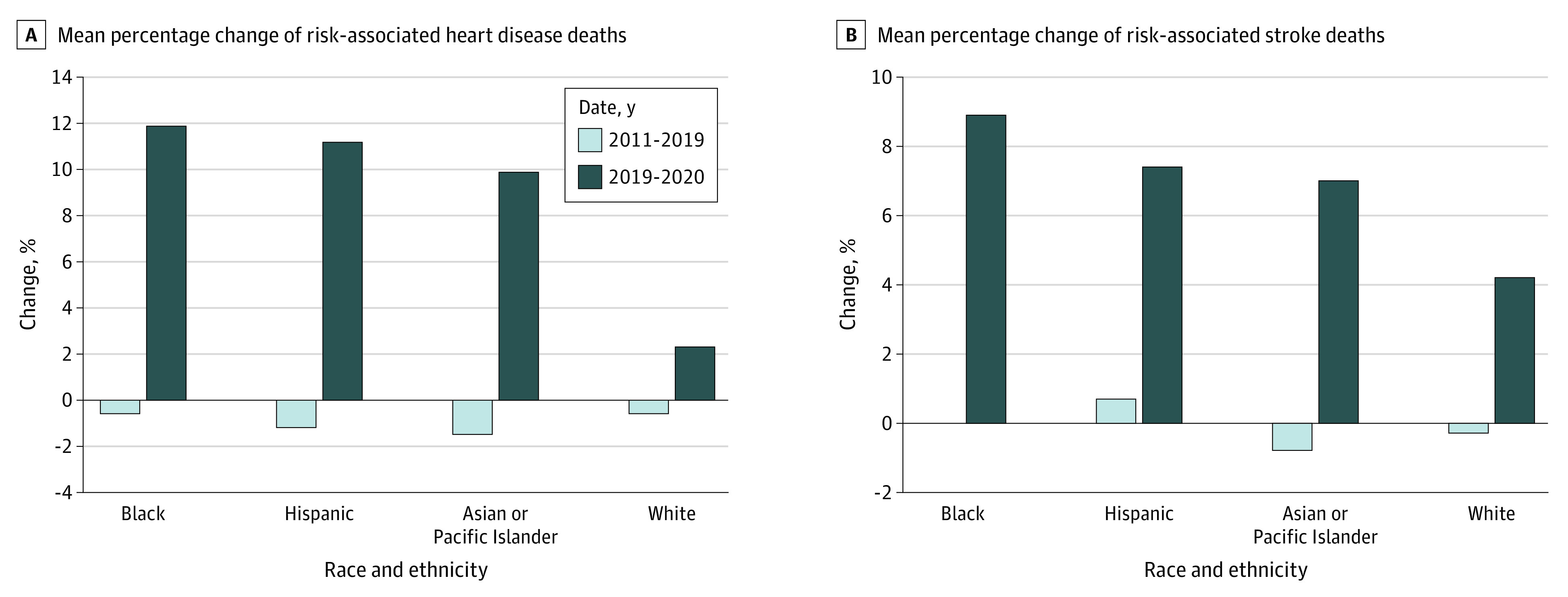
Change in Risk-Associated Heart Disease and Stroke Deaths

The mean annual percent change in number of age-associated HD and stroke deaths from 2011-2019 differed from the percent change from 2019-2020 by less than 1.5% for all race-ethnicity groups. For all race and ethnicity groups, the mean annual percent change in risk-associated HD and stroke deaths from 2011 to 2019 varied from −1.5% to 0.7%, compared with 2019 to 2020 increases ranging from 2.3% to 11.9% (Figure 2). Risk-associated increases were highest in non-Hispanic Black individuals, followed by Hispanic individuals, non-Hispanic Asian or Pacific Islander individuals, and non-Hispanic White individuals, with a more than 5-fold higher percentage increase in non-Hispanic Black individuals compared with non-Hispanic White individuals for HD and a 2-fold higher percent increase for stroke.

## Discussion

This cohort study found an increase in age-associated HD and stroke deaths from 2011 to 2019, while the risk-associated number of HD and stroke deaths decreased. In contrast, from 2019 to 2020, there was a notable several-fold increase in risk-associated deaths from HD and stroke, with greater increases among members of racial and ethnic minority groups, including a concerning highest increase among non-Hispanic Black individuals. A major limitation of this study is potential inaccuracy in coding cause of death.

The COVID-19 pandemic was associated with conditions that likely contributed to risk-associated increased HD and stroke mortality. These conditions included periods of overcrowding of hospitals with patients who had COVID-19, resulting in fewer hospitalizations for acute cardiovascular problems, fewer visits for medical care, poorer medication adherence, and increased barriers to healthy lifestyle behaviors. Complex social determinants are likely associated with increased risk of these diseases.^4^ Our findings, combined with the continuing emergence of new virus strains associated with high COVID-19 rates, suggest that increased emphasis on the maintenance of optimal risk factor levels specified in the American Heart Association’s Life’s Simple 7 guideline^5^ and vigilance toward equity in access to health care are warranted more than ever.
