# Error in Results

**DOI:** 10.1001/jamanetworkopen.2022.11385

**Published:** 2022-04-15

In the Research Letter titled “Age-Adjusted Mortality Rates and Age and Risk–Associated Contributions to Change in Heart Disease and Stroke Mortality, 2011-2019 and 2019-2020,”^1^ published March 23, 2022, there was an error in the Results section. In the second sentence of the first paragraph of the Results section, the values for the total age-associated increase in heart disease deaths covered the period between 2011 and 2019. This article has been corrected.^1^

## References

[1] Sidney S, Lee C, Liu J, Khan SS, Lloyd-Jones DM, Rana JS. Age-adjusted mortality rates and age and risk–associated contributions to change in heart disease and stroke mortality, 2011-2019 and 2019-2020. JAMA Netw Open. 2022;5(3):e223872. doi:10.1001/jamanetworkopen.2022.3872 35319764 PMC8943624

